# Using the theory of constructed emotion to inform the study of cognition-emotion interactions

**DOI:** 10.3758/s13423-022-02176-z

**Published:** 2022-09-09

**Authors:** Gesine Dreisbach

**Affiliations:** Psychology Department, Regensburg University, Regensburg, Germany

**Keywords:** Cognition and emotion, Categorization, Emotion construction

## Abstract

In this article I suggest how theories of emotion construction may inform the study of cognition-emotion interactions. To do so, I adopt the two main concepts *core affect* and *emotions as categories*: Core affect, one’s current affective state, which is defined by the two dimensions pleasure and arousal, is an inherent part of any conscious experience. Specific emotions are understood as categories including highly diverse exemplars. I argue that (1) affective states can and should not be differentiated from cognitive states, and that (2) specific emotions may follow the same principles as other biological or more general categories. I review some empirical evidence in support of these ideas and show avenues for future research.

Psychologists did not invent the concept of 'emotion', for example, to account for certain empirical findings; they obtained certain empirical findings because of their desire to investigate a set of events which their culture had taught them to distinguish as 'emotional'. (Danzinger, 1997; preface p.6)

## Introduction

Cognition-emotion interactions have been a topic of experimental psychology for decades. However, while we have developed many tools and paradigms to study and measure cognitive processes, we still have not found a reliable way to measure emotions in an objective manner. That is, despite the enormous technical development of brain-imaging tools, facial-coding systems, and physiological measures, we still have not found the fingerprint for specific emotions (Barrett, 2017a; Lindquist et al., 2012; Siegel et al., 2018; but see Vytal & Hamann, 2010).^1^ According to the theory of constructed emotion, this, however, is not a failure, but may just never be possible because emotions do not have a unique fingerprint in the brain. Barrett instead suggests that emotions are not automatic and hard-wired reactions to an emotive event but in fact are “the brain’s best guess” based on interoception, past experience, and social context (Barrett, 2017a). That means, theories of constructed emotion (see also Barrett, 2006; Barrett, 2017b; Russel, 2003; Russell & Feldman Barrett, 1999) refute the idea that (biologically hard wired) basic emotions are automatically triggered by an emotive event. Instead, they differentiate between core affect and specific emotions (emotional episodes). Core affect describes the affective state of the conscious mind, which can best be described as a blend of a valence and an arousal dimension. And specific emotions are psychological constructions based on interoceptive stimulation, the individual learning history, and (social) context. Note that, even though I take side in favor of a constructionist view for the sake of the argument, I am of course not denying the existence and relevance of alternative emotion theories.

Here I would like to propose that the theory of constructed emotion may be very helpful to bring some order into the research domain of cognition-emotion interactions. In order to do so, let me begin by shortly introducing the two major concepts of the constructionist view, namely *core affect* and *emotion-as-category* (cf. Barrett, 2006; Russel, 2003). To foreshadow, later I argue that *core affect* primarily modulates *how* humans think, remember, and perceive the world, and that the affective state can in fact hardly be separated from the cognitive state. I then argue that specific *emotion categories* primarily modulate *what* humans think, remember, and perceive. And I show how the emotion-as-category approach informs the study of emotional granularity (the breadth of specific emotions a person can experience) and its role in emotion regulation.

Now, core affect (one’s current affective state), which is defined by the two dimensions valence and arousal, is always there and as such an inherent part of any conscious experience. Feeling more or less pleasant and feeling more or less agitated are inborn features that everybody can report on and which can also be measured objectively from facial, voice, and bodily features (cf. Barrett, 2006, 2017a). Therefore, it should be possible to investigate whether and how an affective state modulates cognition. As outlined below, the literature on the effects of mild positive affect on diverse cognitive processes looks promising in this respect.

The second concept, borrowed from the theory of emotion construction, is the *emotion-as-category* idea (Barrett, 2009, 2017a; Duncan & Barrett, 2007) and needs a broader introduction: Specific emotions are understood as categories, based on the individual learning history, culture, and current context. More precisely, Barrett makes the well-educated suggestion that an emotion (like fear or anger) is a “category that is populated with highly variable instances” (cf. Barrett, 2017b, p.3). Critically, she directly compares emotion categories to any other biological and also more general categories. That means, a category (like fear) resembles a prototype without necessarily implying that such a prototype actually exists. For example, if you were asked to provide a definition of the emotion *fear* you would probably come up with a number of typical features like a pounding heart, trembling, sweating, screaming, freezing, and so forth. But when asked about the last time you experienced fear, you might describe something more or less different from the prototypical description you just provided. The problem with prototypes is, they may be the best example of an (emotion) category, but they by no means need to be the most common (Rosch, 1978/2002; see also Posner & Keele, 1968). It follows that, by definition, the fear that one person experiences may not be comparable to the fear another person experiences (and does not have the same activation pattern in the brain). Moreover, the fear that one person experiences in one moment may be very different (experientially and on the brain level) from the fear that the same person experiences in a different moment. It is therefore not that surprising that the literature on the effects of specific emotions on cognition looks much more diverse because a single specific emotion like for example fear already is so diverse. However, if we take the idea seriously that emotions are activated categories, we might be able to derive testable hypotheses on how specific emotions modulate cognition by looking into the literature on how categories inform perception and action (cf. Barsalou, 1983; Dreisbach, 2012).

But before I outline how core affect and emotion categories modulate cognition, I first need to address a more general problem of how to deal with the unique realness of emotion and cognition on the one side and the lack of a direct correspondence in the brain on the other.

## The mind-brain correspondence problem in the context of cognition and emotion

The question of whether and how to differentiate psychological phenomena like *cognition* and *emotion* is part of a broader discussion on the mind-brain correspondence problem (see Barrett, 2009): While, on a phenomenological level, humans usually are able to differentiate between cognition and emotion, the two cannot be easily distinguished on the brain level. In this context, Duncan and Barrett (2007) talk about observer-dependent psychological categories like memory, thoughts, and emotions that are created by the brain but have no direct correspondence to specific brain structures. That is, only because we have words for a psychological phenomenon does not mean that it has its direct reflection in the brain. Brain-states, by contrast, are observer-independent facts, and their relationship to mind and behavior remains a matter of scientific interpretation. So, how come humans experience a cognitive state differently from an emotional state? Following Barrett, humans are constantly confronted with three sources of information: (1) sensory input from the environment, (2) internal (interoceptive) stimulation, and (3) prior experience. Depending on which source of information is foregrounded at a given moment then determines the psychological state that is experienced as, for example, thought, emotion, or memory (Barrett, 2009; Duncan & Barrett, 2007). This converges with the everyday experience that, when asked about one’s current feeling, humans typically need some time to focus on the relevant information to answer this question. It is important to keep in mind that the observer-dependence of emotion and cognition does not make them less real and an important subject to psychological research. And given their phenomenological (observer-dependent) realness makes it pointless to replace the wording. But acknowledging that cognition and emotion have no direct reference in the brain is important as it changes the study of their mutual influence. This is addressed in the following, focusing on the literature on how (positive) affect and cognition interact.

## Core (positive) affect: The “how”component of cognition-emotion interactions

Positive affect can be understood as one facet of *core affect*. Russel (2003) defines core affect as “a neurophysiological state that is consciously accessible as a simple, nonreflective feeling that is an integral blend of hedonic (pleasure–displeasure) and arousal (sleepy–activated) values” (p. 147). We are not always aware of our current core affect but when asked are able to report on it. To make his point, Russel compares core affect to body temperature: it is always there but you do not think about it all the time. While the average level of positive core affect presumably has a strong genetic component (Lykken & Tellegen, 1996), it can also vary as a function of external factors such as daytime, affective quality of the context, or drug intake. That means that core affect can be transiently altered, as for example by presenting pictures or film clips of a certain affective quality.

Early work by social psychologist Alice Isen and colleagues showed for the first time that positive affect has a systematic effect on cognitive processing styles. In particular, she showed that positive affect increases cognitive flexibility in different cognitive tasks like creative problem-solving, word categorization, and verbal fluency tasks (Isen, 2001; see also Ashby et al., 1999). Since then, tremendous efforts have been made to further the understanding of the underlying mechanisms that account for the increased cognitive flexibility under positive affect. I will not reiterate empirical findings showing how core affect modulates cognition, since there already exist several timely reviews on how especially positive affect modulates attention and perception (Paul et al., 2021; Pourtois et al., 2017; Vanlessen et al., 2016), and cognitive control (Dreisbach & Fröber, 2019; Gable & Dreisbach, 2021; Goschke & Bolte, 2014). However, what I would like to emphasize is that if one accepts that (core) affective states are an inherent part of any conscious experience, it becomes nearly impossible to differentiate an affective from a cognitive state. In fact, Dörner (2002) already suggested that an affective state is defined by a certain cognitive state (varying in selection threshold, resolution level,^2^ and arousal). That means, when you are in a certain cognitive state, then this *is* your affective state, and vice versa. On the downside, this assumption makes it nearly impossible to falsify claims on how positive affect modulates cognition. The reason being that, if the data do not show the respective pattern, this must mean that the participant was not in the respective affective, i.e. cognitive, state (for a related argument, see also Hefer & Dreisbach, 2020).

Moreover, if cognitive and affective (brain) states phenomenologically only differ with respect to the current foregrounding of (internal, external, prior experience) information, as suggested by Barrett and colleagues (Barrett, 2009; Duncan & Barrett, 2007), the assumed linear causation of one cognitive/affective state by the other is no longer tenable. Instead, a probabilistic model according to which a certain (affective/cognitive) brain state changes the likelihood of a transfer to a specific different brain state would seem more appropriate (cf. Barrett, 2009). However, one problem of why it is so hard to overcome the distinction between cognition and emotion (aside from their phenomenological realness) is rooted in the logic of experimental design where we typically investigate causal relationships by manipulating one factor (e.g., emotion) and measure its impact on another factor (e.g., cognition).

Others also recommend overcoming the separation of cognition and emotion and instead promote the idea that the two are inseparably interconnected (Dixon et al., 2017; Hommel, 2019). Empirical support comes from studies swapping the independent (emotion) and dependent (cognition) variables and show that forcing participants into a certain cognitive processing style has a consistent effect on their mood. For example, instead of investigating the effects of mood induction on cognitive flexibility, Akbari Chermahini and Hommel (2012) reversed the logic and started their experiments by confronting participants with divergent or convergent thinking tasks. They found – as predicted – that participants’ mood improved after the divergent thinking task and worsened after the convergent thinking task. Likewise, Mason and Bar (2012) showed that participants who read word lists with progressive associations (i.e., each consecutive word on the list was associated with the former but the association to the first word of the list diminished progressively) rated their mood more positively than after reading a stagnant word list (where all the words were associated with one common theme). This latter effect may also be explained by differences in task difficulty and experienced fluency of processing, both of which are also directly associated with affective experiences (cf. Dreisbach & Fischer, 2015; Reber et al., 2002).

It follows that not only does a change in core affect come along with altered cognitive processing, but cognitive processing likewise is associated with changes in core affect. This further speaks to the idea that the relationship between affect and cognition is not unidirectional but that the two represent two sides of the same medal. Herz et al. (2020) therefore recently suggested so-called overarching “state-of-minds” (SoM), which they define as holistic mental states that guide perception, attention, thought, behavior, and (!) affect. That is, they also suggest overcoming the distinction between cognition and (core) affect. Likewise, Hommel (2019) recently argued more directly that affect and cognition may not be “separable modules of the human mind/brain but two functions that are derived from the same interconnected cognitive/brain dynamics” (p. 5). Put in simple words, “*how you feel is how you think”* equals “*how you think is how you feel.”* From this perspective, it might in fact be helpful to remind oneself from time to time that the answer to the question “How are you doing?” may not only inform about the addressed person’s current affective but also cognitive state of mind.

## Specific emotions as categories: The “what”component of cognition-emotion interactions

In this section I argue that Barrett’s conceptualization of specific emotions-as-categories together with what we know about the shielding function of categories can inform cognition-emotion interactions. In particular, I review selected literature that demonstrates how categories shield us from information that is unrelated to the currently active category by preferential processing of category-congruent information (cf. Dreisbach, 2012). I further argue that the same processes may apply to felt emotion categories. Thus, when considering cognition-emotion interactions, specific emotions inform us on the “what” component, namely what information is preferentially being processed.

Research on cognition-emotion interactions started with so called mood-congruency effects in memory.^3^ In particular, memory encoding and retrieval processes profit from congruence between the current mood/emotion of the participant (e.g., positive vs. negative, elation vs. depression) and the affective quality of the stimulus material. For example, participants in an elated mood remember more positive than negative childhood events whereas participants in a depressed mood would show the reverse pattern (e.g., Snyder & White, 1982). Likewise, participants in a happy mood are better at learning a list of positive words while participants in a sad or angry mood are better at learning a list of negative words (e.g., Bower, 1981). These early studies converge with the idea that core affect “guides cognitive processing according to the principle of mood congruency” (Russel, 2003, p. 149) and show that specific emotions modulate *what* information is preferentially processed.

As stated in the introduction, Barrett (2017b) suggests that an emotion is “a category that is populated with highly variable instances” (p.3). Her core idea here is that any so-called basic emotion can be understood as a statistical (theoretical, that is) value derived from a variety of diverse exemplars of emotional experiences. To make the consequences of this assumption more graspable, let us take a look at what we already know from category-formation. According to the seminal work of Eleanor Rosch (1978/2002), category formation follows the principle of cognitive economy and perceived world structure. That is, a category ideally is broad enough to include exemplars of sufficient similarity but narrow enough to distinguish from exemplars of a different category. Applied to the context of emotions, Barrett has coined the term *emotional granularity*, which describes the extent to which a person is able to differentiate between different emotional experiences and thus categories (Barrett, 2017a). At first sight, it seems that higher emotional granularity violates the principle of cognitive economy and should therefore be avoided. This, however, contrasts with much evidence that a person who can describe their current mood state at a higher resolution level, who masters emotional granularity, is also better at emotion regulation (Barrett et al., 2001; Kashdan et al., 2015; Smidt & Suvak, 2015). In this context, the shielding function of categories comes into play.

### The functional role of categories

**Fig. 1:**
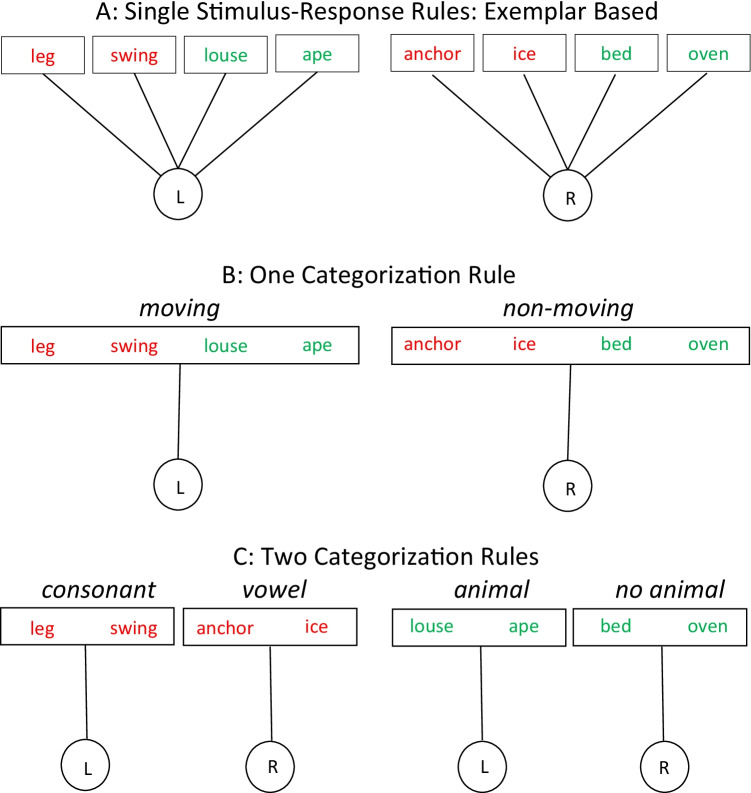
Alternative task representations for the same set of eight-word stimuli that have to be responded to with either a left or right response key (L vs. R). The critical manipulation is how participants are informed about the correct stimulus-response assignment. **a** Participants are told the correct response for each stimulus separately. **b** Participants receive one categorization rule, according to which the stimuli are assigned to two responses. They are told to press the left key, when the word depicts a moving object and the right key if it depicts a static object. **c** Participants receive two categorization rules that depend on the color of the stimulus. Participants are told that, for words written in red, they would have to press the left key when the word starts with a consonant and the right key when it starts with a vowel. For green words, that they would have to press the left key when the word represents an animal and the right key if it is not an animal. Note that stimuli and assigned responses are exactly the same under all task representations, and that color is only a relevant feature when the two categorization rules are applied

In order to study the functional role of categories and rule usage, my colleagues and I have done extensive research comparing performance between exemplar-based and category-based stimulus-response processing (Dreisbach et al., 2007; Dreisbach & Haider, 2008, 2009; Dreisbach & Wenke, 2011; Reisenauer & Dreisbach, 2013, 2014; for a review, see Dreisbach, 2012). To illustrate this reasoning, Fig. 1 depicts potential alternative task representation that were induced by instruction.

The experimental logic is always the same: Participants receive a set of stimuli that have to be responded to with a left or right response key. Depending on whether participants use exemplar-based task representations (i.e., stimulus-response rules) or more or less broad categorization rules, has systematic effects on performance with differential costs and benefits: The obvious benefit of categories is cognitive economy, because one does not have to remember the response assignment for every single exemplar but simply the category-defining stimulus feature and its associated response. Another benefit is that an activated category enhances the processing of any information that is related to this category (i.e., the category animal enhances the processing of features that are related to animals) and thereby shields against potentially interfering unrelated information (Dreisbach & Haider, 2008, 2009). On the downside, this shielding function of categories comes at a cost when the categorization rule changes. Specifically, when two rules are applied (Fig. 1c) a switch between rules incurs switch costs in terms of slower response times (RTs) and/or more errors that cannot be found in the groups that use one rule or exemplar-based (i.e., Stimulus-Response; SR) processing. That is, the usage of categories helps shielding against distraction but also makes the cognitive system less flexible when it is necessary to apply a new category (Dreisbach, 2012; Dreisbach & Wenke, 2011).

Another interesting side-aspect of our findings was that participants were extremely good at forming ad hoc categories (cf. Barsalou, 1983) by themselves when no categories were provided via instruction. In one experiment, we confronted participants with eight different word stimuli and their corresponding (left or right) response. After they had worked through several blocks with these stimuli, we asked participants whether they had remembered the stimulus-response assignments simply by heart or whether they had used another memory strategy. About half of the participants reported having created (ad hoc) categorization-rules that helped them to group the left-response and right-response stimuli, respectively (e.g., “things that I like vs. things that I do not like”; Dreisbach & Haider, 2009; Experiment 2). Interestingly, such ad hoc categories followed the same (shielding) mechanisms as instructed or natural categories (cf. Barsalou, 1983).

### Specific emotions as categories

If specific emotions are (ad hoc) categories, as suggested by Barrett (2017a, 2017b), namely psychological constructs that assign meaning to multi-modal external and internal information in a given moment, then such an emotion category should follow the same principles as cognitive/semantic categories. Note that the term “ad hoc category” was originally coined by Barsalou (1983), who showed that humans are exceptionally prone to forming arbitrary categories in a context-sensitive manner in accordance with current goals (like “things I need when going on a picnic”). By applying this term to emotion categories, Barrett emphasizes that emotion categories can be temporary transient constructions of the brain that give meaning to the multimodal sensory (internal and external) stimulation.

Now, derived from the shielding function of categories, an activated emotion should determine which information is preferentially processed and thereby reduce the impact of information that does not fit into this emotion category. To give an example, let us imagine a person who experiences a specific emotion like anger when entering the commuter train in the morning. The felt anger enhances the processing of information that is related to this emotion (e.g., the face mask of one person in the crowded train that doesn’t cover his nose during a pandemic). This information may even keep the anger emotion activated; now let us imagine a person who experiences a not further differentiated mixture of negative emotions (e.g., “feeling really crappy”) without being able to differentiate between them. This mixture of emotions would be a broader category and therefore enhance the processing of all the information that this feeling is potentially related to. To stick with the example of the commuter train, a crying child, a homeless person, the stranger screaming on his cell phone, an aggressive dog, garbage would all be information related to the currently felt emotion “crappy.” That is, not only anger-related information but also information that is related to sadness, embarrassment, fear, disgust, and so forth would be preferentially processed. It is easy to imagine that the latter person is more prone to persisting in their emotional state because the less differentiated emotion mixture would be confirmed by more (potentially related) information. By contrast, higher emotional granularity, the ability to experience a broader range of specific emotion categories, should make it easier to switch away from the specific emotion because narrower categories find less confirming information and should therefore be less stable. It follows that the shielding function of categories that is supported by preferential processing of information that is related to this category may be one cognitive mechanism that explains benefits and costs of high versus low emotional granularity reported in the literature: Higher granularity enables more flexible emotion regulation because a given emotion gets less support from the environment. Likewise, lower granularity increases the risk of getting stuck in a specific emotion as it gets constantly fueled by supportive information. Such a mechanism may also contribute to the widely observed persistence of depression (e.g., Teasdale, 1988; for a review, see Gotlib & Joormann, 2010).

However, not only the breadth of a given category will influence the range of information that will be processed, but also the strength of the emotion felt. And, again derived from what we know of the shielding function of categorization rules, the stronger a specific emotion is activated/experienced, the harder it will be to “switch away” from this category because information that is not related to that emotion but could potentially change the currently felt emotion has less impact (Reisenauer & Dreisbach, 2013, 2014). Interestingly, a recent EEG study suggests that participants with higher emotional granularity (measured a day before the lab experiment) show more differential processing for pictures belonging to different specific emotions (Lee et al., 2017). This could be taken as the first indication that higher emotional granularity in fact goes hand in hand with more emotion specific processing on the neuronal level. However, there might be a limit where higher emotional granularity turns into a disadvantage. Imagine a person experiencing emotions that are as fine grained as described by William James, who compared the “entire organism … as a sounding-board, which every change of consciousness, however slight, may make reverberate” (James, 1890/2012, chapter 25). One can only imagine how exhausting it would be for someone who actually feels and experiences these subtle changes as emotional. I would therefore assume that emotional granularity reaches its limits when the resolution level becomes too high.

### Does the shielding function of (emotion) categories depend on emotion construction?

One may ask whether the shielding function of (emotion) categories necessarily relies on a constructionist view of emotions. To answer this question, it is important to understand how emotions, according to Barrett’s constructionist view, emerge (or rather: are created by the brain): The (conscious) brain is constantly confronted with internal (bodily) and external stimulation, and it uses past experiences and knowledge to predict and give meaning to this ongoing sensory input. Depending on the foregrounding of internal sensations, an emotion is then constructed/experienced (Barrett, 2009; Hoemann & Barrett, 2019; Hutchinson & Barrett, 2019). Barrett and colleagues talk about “situated conceptualizations” in this context, to highlight the fact that the brain – in any awake moment – assigns meaning to the multimodal sensory input (Barrett et al., 2014). An emotion emerges when the sensory input is foregrounded and shares enough similarities with prior experiences of the said emotion. A child, who does not have the respective emotional words to describe its feelings (who is experientially blind in this respect), relies on the interpretation of its caregivers and thereby acquires (emotional) knowledge that then can be used to give meaning to future sensory events. That is, just as the child learns to differentiate cats from dogs, it will learn to differentiate fear from anger (Hoemann et al., 2019).

Acquiring emotional knowledge does not stop in infancy and can even be trained by short-time intervention as a recent study suggests: Vedernikova et al. (2021) showed that a short-term training to increase emotional knowledge led to higher emotional granularity for negative emotions, even lasting 1 month after the intervention. Given that people with higher emotional granularity are better at emotion-regulation and less likely to be overwhelmed by distress (Kashdan et al., 2015), it seems important to find ways to improve emotional granularity and further the understanding of the so far not well understood mechanisms that account for the beneficial effects. The constructionist view of emotions therefore has implications for increasing emotional granularity and – consequently – improving emotion regulation. And it implies that maladaptive (learnt) emotions could be “deconstructed” by training and be replaced (i.e., regulated) by ad hoc (emotion) categories. And here is where the shielding function of categories comes into play again: A potential emotional training could for example assign different features of two different but easily confused specific emotions (e.g., nervousness vs. excitement) to two responses and either inform participants upfront about the underlying emotion categories (category-based processing) or not (exemplar-based processing). Those using categories should be less distractible by stimulus features that do not belong to the currently active emotion category and also be better at differentiating between said emotions in a later emotion-rating test.

## Avenues for future research

As outlined above, core affect together with the emotion-as-category idea opens new avenues for the study of cognition-emotion interactions. First of all, with respect to the assumed equivalence of affective and cognitive states in the context of core affect, more empirical evidence is needed. To this end, further studies should be conducted that swap the dependent and independent measures of traditional experiments on cognition-emotion interactions by manipulating the cognitive state and measuring its impact on the affective state. But research should not stop here. As outlined above, it seems neither possible nor desirable to deny the psychological realness of cognition and emotion as distinct phenomenological states; but given the lack of a direct correspondence in the brain, what we need to understand is how the process of internal versus external foregrounding (Barrett, 2009; Duncan & Barrett, 2007; Hoemann & Barrett, 2019) takes place that then gives rise to the individual experience of emotion or thought. One potential first step to approach this subject would be to explicitly manipulate the foregrounding of either internal or external stimulation (all else being equal) and to measure its impact on behavior and subjective experience.

When it comes to emotion-as-category, this idea may inform the study of emotion development, emotion regulation, and emotion dissipation:

### Emotion development in children

Kharitonova and colleagues investigated abstract rule usage in 3-year-old children using a card sorting task. They found that only those who were able to apply a new rule to the same set of cards were also able to generalize this rule to entirely new cards (Kharitonova et al., 2009). On a neuronal level, this ability presumably develops in synchrony with the later maturing dorsolateral and rostrolateral prefrontal cortex (cf. Bunge & Zelazo, 2006; see also Badre, 2008). Interestingly, the development of category usage falls together in time with the development of specific emotions. And irrespective of how exactly infant emotion concepts develop (for a recent discussion, see Ruba & Repacholi, 2020; Hoemann et al., 2020), the emotion-as-category approach suggests that it may be worthwhile to investigate the relation of abstract rule usage, emotional granularity, and emotion control in children.

### Emotion regulation and dissipation

There already exist valuable approaches and methods to train strategies of emotion regulation in a non-clinical context (Gross, 1998; for a taxonomy, see Koole, 2009). As already outlined above, the shielding function of categories that is supported by the preferential processing of any information that is related to the currently activated (emotion) category may explain the persistence of broader and highly activated categories. A dysphoric person may find more information that potentially fits into the negative emotion category, and it may also bias the interpretation of the surrounding information in favor of the activated category (e.g., Gotlib & Joormann, 2010). Interestingly, training the ability to form ad hoc categories appears to improve creativity and flexibility in a later problem-solving task (Chrysikou, 2006). One interesting question for future research would therefore be how training in category formation (emotional and non-emotional) may improve emotional granularity and eventually higher flexibility in emotion regulation (Aldao et al., 2015). More precisely, training a dysphoric participant to apply different categorization rules to features belonging to different negative and positive emotions might promote a more granular processing. Given the recent evidence that an intervention that targets increasing emotion knowledge has already proven successful in increasing emotional granularity (at least for negative emotions) sounds promising in this respect (Vedernikova et al., 2021).

## Conclusion

Theories of emotion construction differentiate between core affect and specific emotions. Here I have argued that one’s current core affect may not be structurally different from one’s cognitive state such that a change of one state necessarily will change the other. From the traditional view of cognition-emotion interactions, the affective state determines *how* information is being processed. From the stand taken here, the cognitive state would likewise determine one’s core affect (*how* you feel in a given moment). Specific emotions – seen as ad hoc categories – determine *what* is being processed in a given moment. The shielding functions of categories thereby may provide the potential underlying mechanism for the beneficial effects of emotional granularity reported in the literature (the preferential processing of emotion congruent information). But it also allows us to derive a new and testable hypothesis – for example, how the ability to form new ad hoc (emotion) categories might modulate emotion regulation in children and adults.

## Footnotes

1 I am aware of the ongoing debate between the different “emotion camps” ranging between basic inborn emotions and theories of emotion construction. The aim of this article is not to discuss the pros and cons between both sides, but instead to lay out how the idea of emotions as categories, as most prominently defended by Lisa Feldman Barrett, would inform cognition-emotion interactions.

2 Dörner defines *selection threshold* as the motivation strength of a currently active intention that inhibits concurrent intentions and goals. *Resolution level* is defined as the breadth of associations within a neural network; it depends on the strength of mutual inhibition within this network.

3 Even though here I make the distinction that core affect primarily modulates the cognitive processing style (and vice versa), whereas specific emotions modulate *what* is being processed, mood-congruency effects can be found for both core affect and more specific emotions.
